# Lazy Approaches for Interval Timing Correlation of Sensor Data Streams

**DOI:** 10.3390/s100605329

**Published:** 2010-05-27

**Authors:** Kiseong Lee, Chan-Gun Lee

**Affiliations:** Department of CSE, Chung-Ang University, 221 Heukseok, Dongjak, Seoul, Korea; E-Mail: goory00@gmail.com

**Keywords:** sensor, interval, lazy, timestamps, correlation

## Abstract

We propose novel algorithms for the timing correlation of streaming sensor data. The sensor data are assumed to have interval timestamps so that they can represent temporal uncertainties. The proposed algorithms can support efficient timing correlation for various timing predicates such as deadline, delay, and within. In addition to the classical techniques, lazy evaluation and result cache are utilized to improve the algorithm performance. The proposed algorithms are implemented and compared under various workloads.

## 1. Introduction

Wireless sensor networks are composed of sensors, embedded computers, and communication devices. They can harvest various interesting information such as light, motion, proximity, temperature, and chemical conditions. There are many emerging applications utilizing the information from sensors. The applications range from simple monitoring systems to sophisticated systems making critical decisions based on the automated analysis of the sensor data.

In this paper, we propose novel algorithms for the timing correlation of streaming sensor data. The sensor data are assumed to have interval timestamps so that they can represent temporal uncertainties. The proposed algorithms can support efficient timing correlations for various timing predicates such as deadlines and delays. The timing correlation enables the users to extract pairs of streaming data, of which sources are different, satisfying specific timing conditions.

In some cases, the timestamp of the data from a sensor cannot be modeled as a scalar value. There can be various reasons such as inactivity of a sensor due to its battery limitation, granularity difference between heterogeneous sensors, and inaccurate timing behavior of a sensor. In addition, the possibility of temporal uncertainty is high because the unexpected hardships can easily happen due to the harsh environments where sensors operate.

In order to capture the timing uncertainty of the timestamp, adopting a time interval as the timestamp is a common approach [1]. Typically, an interval timestamp is composed of two end-time points confining the possible occurrence time. In this study we assume that the probability distribution in the interval is uniform. In other words, any time point in the interval is assumed to be equally probable.

In our previous work, we designed an efficient algorithm for the timing correlation by analyzing the upper-bounds and lower-bounds of the satisfaction probability on time intervals.

We further extend the algorithm by adopting the approaches of the lazy evaluation and the result look-up in this study. The extended algorithms show better performance by exploiting unveiled properties of timing correlations presented in this paper. We implement various timing correlation algorithms and compare them under various workloads.

Main contributions made in this paper are shown in the following:

- Extending the algorithm by adopting a lazy evaluation approach: We extend the previously designed algorithm by adopting a lazy approach and correlating the sensor data by the blocks.
- Extending the algorithm by adding the look-up technique: In order to avoid expensive calculation for satisfaction probability in probe regions, we add an idea of look-up technique based on new observations of the timing correlation.

The rest of this paper is organized as follows. Section 2 presents related work. In Section 3, we present an overview of timing correlations for sensor data. A brief review of the timing correlation is given. Section 4 presents a review of previously studied algorithms and designs extended versions of the timing correlations. In Section 5, we compare the performance of the algorithms under various workloads and analyze the results. Section 6 presents the future work and summary.

## 2. Related Work

There have been many studies on the sensor data processing in recent years. One of the most active research areas related to the sensor data processing is stream data management systems (SDMSs). Babcock *et al.* [2] and Golab and Ozsu [3] present extensive surveys on stream data processing and recent advances.

Since we are interested in the timing correlation problem, we shall restrict our discussion to the problem of the correlation of stream data.

In [4], a box figure is used to represent a stream query operator and a link between the boxes connects the output port of a box to the input port of another box. The users can express their queries by arranging the query boxes and the links. The join operators typically have parameters including join predicates, time window sizes, and ordering information about the streams. The ordering information specifies the tolerance of disorder.

Hammad *et al.* [5] presents BEW-join and FEW-join which are variants of classical sliding window joins. One of the different assumptions made in our paper is about the arrival ordering of streaming data. Hammad assumes that the streaming data are delivered to the system in timestamp-sorted order. In other words, there is no out-of-order data. They present efficient join algorithms by utilizing this assumption. Our proposed techniques can handle the cases where there exist out-of-order data.

There are studies based on the cost analysis of the sliding window joins. Kang *et al.* [6] present a unit-time basis cost model for the sliding window joins and propose efficient join strategies based on the analysis. In addition, effective resource-allocation schemes for improving the efficiency are proposed.

In order to handle the streaming data arriving in out-of-order, Srivastava and Widom [7] propose the use of heartbeats in the stream processing. The meaning of a heartbeat with the timestamp *τ* from a stream is that there will be no future data with timestamp less than *τ* from the stream.

Recently Wu *et al.* [8] propose efficient techniques for the memory management in the context of the stream join. They point out that the query-driven method is not aware of the input characteristics, thus the data-driven approach, Window-Oblivious Join, is needed.

None of the above work address the cases of the interval timestamp. As stated in Introduction, the timestamp of a streaming data from sensors may have temporal uncertainties. In order to handle this inherent uncertainties in timestamps, we adopt interval timestamps and assume that the probability distribution in a given interval is uniform.

Dyreson and Snodgrass [1] present solutions in the context of valid-time databases where a data is accompanied with a interval timestamp during which the data is valid. They take a probabilistic approach like ours in order to cover indeterminacy in timestamps. An algorithm for computing the satisfaction probability of the comparison operator *before* for interval timestamps is presented.

Our earlier work [9] address the problem of calculating the satisfaction probability of timing predicates on interval timestamps. A timing predicate is defined over interval timestamps with a deadline/delay. A timing condition is composed of a timing predicate and its satisfaction probability threshold. We extended [9] to include the pruning algorithms which are useful in monitoring time critical systems in [10]. [11] focuses on the problem of interval timing correlation. By utilizing the analysis results of the satisfaction probability, it provides efficient techniques for interval timing correlation. In this paper, we further extend the algorithms in [11] by adopting the approaches of the lazy evaluation and the result look-up.

## 3. Timing Correlation for Sensor Data

In this section, we present the problem of the interval timing correlation and review the main findings discussed in the previous studies. Sensors measure and transmit data to harvesting facilities. The harvesting facilities can be another typical sensors or specialized devices. Finally, the data are sent to a system (or systems for distributed computing) taking the role of the data analysis.

In general the sensor data processors filter out unnecessary data and forward a subset of the data which may be useful to the next data processors for further analysis. One of the typical operators used during this phase is the timing correlation. The timing correlation operators allow us to collect pairs of data which are satisfying a predefined timing condition. For example, a user may want to extract pairs of data such that the time differences of the pairs are within 5 seconds.

Specifically, we are interested in the timing correlation operator which can handle the interval timestamps. In order to specify a timing condition over interval timestamps, we take a probabilistic approach. The users of the system present an interval timing correlation by defining a timing predicate on interval timestamps. The timing predicate can be a form of deadline or delay.

A deadline constraint requires that a corresponding event should happen provided that a triggering event happen before the timer accompanied with the constraint expires. Assume that there exists a deadline constraint with a specific time *t* where *e*_1_ is a triggering event and *e*_2_ is the corresponding event. If there is another deadline constraint with the same time *t* where *e*_2_ is a triggering event and *e*_1_ is the corresponding event. In that case, we state that a mutual deadline constraint is defined on the events *e*_1_ and *e*_2_. The mutual deadline is the most popular timing predicate in interval timing correlations. Hence, most of our examples will be the mutual deadlines.

A delay constraint requires that the corresponding event should not happen provided that a triggering event happen before the specified time passes.

An interval timing correlation requires a confidence threshold, which determines the minimum satisfaction probability of the timing predicate. Typically the interval timing correlation operator produces streams of paired data which satisfy the given interval timing correlation condition.

In this paper, we adopt the event model proposed in [9]. We shall briefly explain the event model in the following. The timestamp is composed of a pair of time values (*min_time*, *max_time*) where min_time represents the earliest time of the event occurrence. The latest time is represented by the max_time. We assume that the probability distribution in the timestamp is uniform.

In addition, the following notations are used in the remaining of this paper. For a timestamp *I* = (*min_time*, *max_time*), **min(I)** and **max(I)** extracts the *min_time* and *max_time*, respectively. **length(I)** returns the value *max*(*I*) − *min*(*I*). *π* and *ρ* represents the longest and the shortest possible length of any timestamp. The parameters should be fixed in system design time. **Satisfaction probability** *prob*(*tp*) of a timing predicate *tp*, is the probability for which *tp* is satisfied. The pair of *tp* and *ct* (*tp*,*ct*) is called a **timing condition**, where *tp* is a timing predicate and *ct* is a confidence threshold requirement for *tp*. We define that a timing condition (*tp*, *ct*) is satisfied if *prob*(*tp*) ≥ *ct* and it is violated if *prob*(*tp*) < *ct*.

In the remainder of this paper, we shall use the symbol “@” to indicate the timestamp of a tuple. If the symbol is used in front of a stream name, then it means the timestamp of any tuple sent from the stream. For example, @*e* represents the timestamp of the tuple *e*. The timing predicate |@*S*_1_ – @*S*_2_| ≤ *d* means that we want to extract a pair of (*e*_1_, *e*_2_) satisfying the mutual deadline *d* where *e*_1_ and *e*_2_ are tuples from stream *S*_1_ and *S*_2_ respectively.

We derived formula for calculating the satisfaction probabilities of the deadline and delay predicates in our previous work [9].

**Theorem 1** *[9] Given a deadline predicate, c : I*_1_ + *d* ≥ *I*_2_ *where d* ≥ 0*, the satisfaction probability of c, prob*(*c*) *can be computed by the following expression:*

$$\mathrm{prob}(c)=\frac{1}{\mathrm{len}(I_{1})\mathrm{len}(I_{2})}\;\int_{\min(I_{1})}^{\max(I_{1})}\mathrm{MIN}(\mathrm{MAX}(x+d-\min(I_{2}),\;0),\;\mathrm{len}(I_{2}))\mathrm{dx}$$

The satisfaction probability of a delay predicate can be derived similarly.

In this paper, we assume that any deadlines specified in timing predicates are larger than *π* the maximum interval length in the system. Similarly any delays are smaller than −*π*, which means |*d*| ≥ |*π*|. This assumption makes the computation of the mutual deadline predicates simple as shown in the following:

**Corollary 1** *[11] Given a mutual deadline predicate, c :* |*I*_1_ − *I*_2_| ≤ *d, prob*(*c*) *= prob*(*I*_1_ + *d* ≥ *I*_2_) *if min*(*I*_1_) ≤ *min*(*I*_2_) *and* |*d*| ≥ |*π*|.

The computation of the satisfaction probability of a deadline constraint can be simplified by categorizing the problems into six different cases based on the relations of *I*_1_ + *d* and *I*_2_. Interested readers are invited to our previous study in [10] for details.

## 4. Design of Timing Correlation Operators

### 4.1. Efficient Timing Correlation

Throughout this section, we assume that there is an interval timing correlation such that it has a mutual deadline for two events such as |@*S*_1_ − @*S*_2_| ≤ *d* and a confidence threshold *ct*. In other words, we want to extract a pair of (*e*_1_, *e*_2_) satisfying the mutual deadline *d* and their satisfaction probability should be at least *ct*.

**Figure 1.**
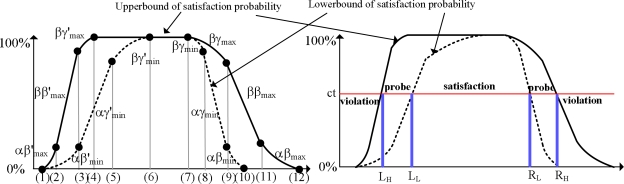
(a) The upper-bounds and the lower-bounds of satisfaction probabilities (b) Efficient filtering process using the bounds.

Figure 1 illustrates the core results studied in [11]. In the figure, we assume that a base tuple with the timestamp *I*_1_ = (*min*_1_, *max*_1_) has arrived from stream *S*_1_. The graph presents the upper-bounds (solid lines) and the lower-bounds (dotted lines) of satisfaction probabilities for each possible *x* = *max*(*I*_2_) where *I*_2_ is the timestamp of a tuple in the targets stream (*S*_2_ in this specific example).

A timing correlation process starts upon receiving a tuple from a stream. The tuple and the stream is referred to as *base tuple* and *base stream* respectively. The other stream is called as *target stream*.

By using the information shown in the Figure 1(a), we can efficiently partition the target tuples in order to extract the results satisfying the timing correlation as listed in the following:

1. Any target tuple with the timestamp *I* where *max*(*I*) ∈ [*L_L_*, *R_L_*] is guaranteed to satisfy the given timing condition.
2. Any target tuple with the timestamp *I* where *max*(*I*) < *L_H_* is guaranteed to violate the given timing condition.
3. Any target tuple with the timestamp *I* where *max*(*I*) > *R_H_* is guaranteed to violate the given timing condition.
4. Any target tuple with the timestamp *I* where *max*(*I*) ∈ [*L_H_*, *L_L_*) needs an evaluation of the satisfaction probability.
5. Any target tuple with the timestamp *I* where *max*(*I*) ∈ (*R_L_*, *R_H_*] needs an evaluation of the satisfaction probability.

where *L_L_*, *R_L_*, *L_H_*, *R_H_* are the X-axis values of the points crossing with a horizontal line of which Y-axis value is *ct* as shown in Figure 1(b). *ct* is the confidence threshold requirement of the timing condition.

From the figure, the above observations are intuitively derived. For example, any target tuple with the max(timestamp) ∈ [*L_L_*, *R_L_*] is guaranteed to satisfy the given correlation condition; its minimum satisfaction probability must be greater than or equal to the requested confidence threshold. In our previous work [11], we presented efficient algorithms for performing interval timing correlations by using the above result. In the next subsection, we extend the previous findings and present two new algorithms for interval timing correlations.

### 4.2. Algorithms for Interval Timing Correlation

In this section, we review the algorithms for the interval timing correlation proposed in [11] and introduce novel algorithms.

The *simple timing correlation* is the most simplest one among the algorithms discussed here. When a tuple *e* arrives at the base stream, every tuple in the target stream buffer is examined and the satisfaction probability is calculated. While the system is visiting the tuples in the target stream buffer, it marks the obsolete tuples. Finally the marked tuples are removed from the target stream buffer and *e* is inserted into the base stream buffer.

**Algorithm 1:** SimpleTimingCorrelation(*e_new_*, BaseStream)

1:	**for all** tuple *e* in the target buffer **do**
2:	**if** (*ct* ≤ *prob*(|@*e_new_* − @*e*| ≤ *d*)) **then**
3:	Add (*e_new_*, *e*) to the result
4:	**end if**
5:	Mark *e* if it is obsolete.
6:	**end for**
7:	Remove the marked obsolete tuples in the target buffer.
8:	Insert *e_new_* into the end of base buffer.

The Simple-Sort (SSort in short) timing correlation slightly modifies the simple timing correlation such that it keeps the tuples in order with respect to the max timestamps. Hence, the algorithm expects longer blocks of obsolete tuples consecutively located than those in the simple timing correlation.

The *eager timing correlation* uses the upper-bounds and lower-bounds of the satisfaction probability presented in the previous section. Every time a tuple *e* arrives, the system computes *L_H_*, *L_L_*, *R_L_* and *R_H_* based on *e*. As illustrated in the previous section, all tuples belonging to [*L_L_*, *R_L_*] in the target stream buffer are guaranteed to be in the correlation result. The tuples belonging to [*L_H_*, *L_L_*) or (*R_L_*, *R_H_*] in the target stream buffer should be probed further. To determine the block of invalid tuples, we first set @*e_inv_* to (*CurrentTime* − *delay_base_* − *π, CurrentTime* − *delay_base_*) where *delay_base_* is the maximum delay in the base stream. Then, we compute *L_H_* based on *e_inv_*. The target tuples having the timestamp *I* such that *max*(*I*) < *max*(*L_H_*(*e_inv_*)) in the target buffer are guaranteed not to be in the result with any future incoming base tuples. Hence, they can be removed from the target buffer.

**Algorithm 2:** EagerTimingCorrelation(*e_new_*, BaseStream)

1:	Compute *L_H_*, *L_L_*, *R_L_*, and *R_H_* based on *e_new_*
2:	**for all** tuple *e* belongs to [*L_L_*, *R_L_*] in the target buffer **do**
3:	Add (*e_new_*, *e*) to the result
4:	**end for**
5:	**for all** tuple *e* belongs to [*L_H_*, *L_L_*) or (*R_L_*, *R_H_*] in the target buffer **do**
6:	Probe(*e_new_*, *e*, *d*, *ct*)
7:	**end for**
8:	Invalidate obsolete tuples in the target buffer by *L_H_* (*e_inv_*).
9:	Insert *e_new_* into the base buffer in a sorted order.

**Algorithm 3:** Probe(*e_new_*, *e*, *d*, *ct*)

1:	**if** ct ≤ prob(|@*e_new_* − @*e*| ≤ *d*) **then**
2:	Add (*e_new_*, *e*) to the result
3:	**end if**

The *lazy timing correlation* also uses the upper-bounds and the lower-bounds like the eager algorithm; however, it does not start correlation processing every time a new tuple arrives. Upon receiving a new tuple, the lazy timing correlation just inserts the tuple into the appropriate stream buffer and waits until its re-evaluation time [12].

When to re-evaluate can be determined either by the number of unprocessed tuples or by a time frequency (or both). For example, a system can be designed to re-evaluate whenever there are more than 500 unprocessed tuples or every one second.

It is noted in [12] that the lazy correlation is preferable over the eager correlation when the arrival rates of data streams are so high that it is hard to handle the incoming every tuple instantly.

However, the benefit of the lazy algorithm comes at the expense of the longer response time; until the re-evaluation condition is met, the already arrived but un-evaluated tuples should wait in the buffers. Therefore, the re-evaluation condition must be designed carefully not to violate the system performance requirements. The algorithm for the lazy timing correlation is presented in Algorithms 4 and 5.

**Algorithm 4:** LazyTimingCorrelation(*e_new_*, BaseStream)

1:	Insert *e_new_* into the end of base buffer.
2:	**if** there are “enough” tuples **then**
3:	call BlockTimingCorrelation(BaseStream)
4:	**end if**

**Algorithm 5:** BlockTimingCorrelation(BaseStream)

1:	Sort the target stream buffer
2:	*tp_l_* ← the first tuple in the unprocessed block in the base stream buffer
3:	*tp_r_* ← the last tuple in the unprocessed block in the base stream buffer
4:	**for***e_new_* = *tp_r_* to *tp_l_* in the base stream buffer **do**
5:	Compute *L_H_*, *L_L_*, *R_L_*, and *R_H_* based on *e_new_*
6:	**for all** tuple *e* in [*L_L_*, *R_L_*] in the sorted block of the target stream buffer **do**
7:	AddResult(*e_new_*, *e*)
8:	**end for**
9:	**for all** tuple *e* in [*L_H_*, *L_L_*) or (*R_L_*, *R_H_*] in the sorted block of the target stream buffer **do**
10:	Probe(*e_new_*, *e*, *d*, *ct*)
11:	**end for**
12:	**end for**
13:	Sort the base stream buffer
14:	Invalidate obsolete tuples in the base buffer by *L_H_* (*e_inv_*).
15:	Invalidate obsolete tuples in the target buffer by *L_H_* (*e_inv_*).

Now we extend the lazy timing correlation to use look-up tables in order to perform the probing process more efficiently. The following corollary presents properties used in the algorithm.

**Corollary 2** *Assume there are timestamps I*_1_*, I_i_, and I_j_* *where max*(*I*_1_) ≤ *max*(*I_i_*) ≤ *max*(*I_j_*) *and min*(*I*_1_) ≤ *min*(*I_i_*) ≤ *min*(*I_j_*). *If a timing condition (I*_1_ + *d* ≥ *I_j_*, *ct) is satisfied then, so is the timing condition (I*_1_ + *d* ≥ *I_i_*, *ct). Similarly if a timing condition (I*_1_ + *d* ≥ *I_i_*, *ct) is violated, then so is (I*_1_ + *d* ≥ *I_j_*, *ct).*

**Figure 2.**
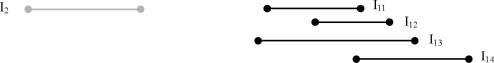
Efficient probing example.

**Example 1** *Assume there are timestamps as shown in Figure 2. Suppose that (I*_2_ + *d* ≥ *I*_11_, *ct) is violated. Then, by Corollary 2, we can claim that (I*_2_ + *d* ≥ *I*_12_, *ct) and (I*_2_ + *d* ≥ *I*_14_, *ct) are also violated without computing the satisfaction probabilities. Now suppose that (I*_2_ + *d* ≥ *I*_12_, *ct) is satisfied. Then, by Corollary 2, we can claim that (I*_2_ + *d* ≥ *I*_11_, *ct) is also satisfied.*

The main idea of the extended algorithm is to reuse the satisfaction probabilities calculated in the probe regions. As illustrated in the previous example, while performing an interval timing correlation for two blocks of tuples, there can be cases where we can reuse the previous calculation results and avoid expensive probability computations. By comparing Algorithm 6 and the lazy timing correlation, we can notice that the main difference is the way of handling probe regions. To process the probe regions, the algorithm first initializes the look-up table after identifying the range of target tuples. There are two probing regions but their processes are symmetric; hence we shall explain only the part handling the left probe region.

**Algorithm 6:** LazyWithLookup-newblock(BaseStream)

1:	Sort the target stream buffer
2:	*tp_l_* ← the first tuple in unprocessed block in the base stream
3:	*tp_r_* ← the last tuple in unprocessed block in the base stream
4:	**for***e_new_* = *tp_r_* to *tp_l_* in the base stream buffer **do**
5:	Compute *L_L_*, *R_L_* based on *e_new_*
6:	**for all** tuple *e* belongs to [*L_L_*, *R_L_*] in the sorted block of the target buffer **do**
7:	Add (*e_new_*, *e*) to the result
8:	**end for**
9:	**end for**
10:	*leftindex* ← the index for *L_H_*(*max*(@*tp_l_*) – *π*, *max*(@*tp_l_*)) in the target stream buffer
11:	*rightindex* ← the index for *L_L_*(*max*(@*tp_r_*) – *ρ*, *max*(@*tp_r_*)) in the target stream buffer
12:	Initialize look-up table (*leftindex*, *rightindex*)
13:	**for***e_new_* = *tp_r_* downto *tp_l_* in the base stream buffer **do**
14:	Compute *L_H_*, *L_L_*, based on *e_new_*
15:	**for all** tuple *e* belongs to [*L_H_*, *L_L_*) in the target buffer **do**
16:	EfficientProbe(*e_new_*, *e*, *d*, *ct*, *BaseStream*)
17:	**end for**
18:	**end for**
19:	Initialize look-up table (*leftindex*, *rightindex*)
20:	**for***e_new_* = *tp_l_* to *tp_r_* in the base stream **do**
21:	Compute *R_L_*, *R_H_*, based on *e_new_*
22:	**for all** tuple *e* belongs to (*R_L_*, *R_H_*] in the target buffer **do**
23:	EfficientProbe(*e_new_*, *e*, *d*, *ct*, *BaseStream*)
24:	**end for**
25:	**end for**
26:	Invalidate obsolete tuples in the base buffer by *L_H_* (*e_inv_*).
27:	Invalidate obsolete tuples in the target buffer by *L_H_* (*e_inv_*).

The algorithm traverses the base tuples in the unprocessed block in reverse chronological order. Once we computed prob(|@*e_b_* – @*e_t_*| ≤ *d*) where *e_b_* is a tuple in the base stream and *e_t_* is a tuple in the target stream, we store the timestamp of *e_b_* as well as the computed satisfaction probability into the look-up table. When we compute prob(|@*e*′*_b_* – @*e_t_*| ≤ *d*) where *e*′*_b_* is another tuple in the base stream, we first check whether *e*′*_b_* can use the result computed based on *e_b_*. This decision is made by comparing the min values of @*e_b_* and @*e′_b_*. If *min*(@*e*′*_b_*) ≤ *min*(@*e_b_*), then it means that @*e*′*_b_* is strictly less than @*e_b_*. (We define *e*′*_b_* is strictly less than *e_b_* iff *min*(@*e*′*_b_*) ≤ *min*(@*e_b_*) and *max*(@*t*′*_b_*) ≤ *max*(@*t_b_*).) The algorithm traverses the base stream buffer from the tail; hence, it is trivially true that *max*(@*e*′*_b_*) ≤ *max*(@*e_b_*). In case the @*e*′*_b_* is strictly less than *e_b_*, we can reuse this result; if the stored value is larger than *ct* (the confidence threshold for this timing condition), then it should be the case prob(|@*e*′*_b_* – @*e_t_*| ≤ *d*) is no less than *ct*. Hence, the tuple pair (*e*′*_b_, e_t_*) must be in the final result. Even if the stored value is less than *ct*, it is still possible that (*e*′*_b_*, *e_t_*) can satisfy the interval timing condition. So, we compute the satisfaction probability for (*e*′*_b_*, *e_t_*) and store the new result into the look-up table. If *e*′*_b_* is not strictly less than *e_b_*, then we cannot reuse the stored result; we compute the satisfaction probability and store it.

Recall that the primary purpose of using the look-up table is to avoid the “relatively” expensive operation—the satisfaction probability calculation incurring floating-point operations. To minimize the overhead for accessing to the look-up tables, we used array data structure to implement the look-up table. Hence every access to the look-up table was done via the index to an element in the array.

**Algorithm 7:** EfficientProbe(*e_new_*, *e*, *d*, *ct*, *BaseStream*)

1:	**if** the result in the look-up table is usable **then**
2:	*c* ← lookup(*e*)
3:	**if***c* = *notInit***then**
4:	Probe(*e_new_*, *e*, *d*, *ct*)
5:	SetLookup(prob(|@*e_new_* – @*e*| ≤ *d*), *e*)
6:	**else**
7:	**if***c* ≥ *ct***then**
8:	Add (*e_new_*, *e*) to the result
9:	**else**
10:	Probe(*e_new_*, *e*, *d*, *ct*)
11:	**end if**
12:	**end if**
13:	**else**
14:	Probe(*e_new_*, *e*, *d*, *ct*)
15:	SetLookup(prob(|@*e_new_* – @*e*| ≤ *d*), *e*)
16:	**end if**

Now let us prove the correctness of the look-up technique in the algorithm.

**Corollary 3** *Suppose there are two timestamps I*_1_ *and I*_2_. *Assume that min*(*I*_1_) ≤ *min*(*I*_2_) *and max*(*I*_1_) ≥ *max*(*I*_2_), *i.e., I*_1_ *covers entire I*_2_. *Then prob*(|*I*_1_ − *I*_2_| ≤ *d*) = 1.

Proof:

Since *d* ≥ *π*, *min*(*I*_1_) + *d* ≥ *max*(*I*_1_). By the assumption, *max*(*I*_1_) ≥ *max*(*I*_2_). Therefore, *min*(*I*_1_) + *d* ≥ *max*(*I*_2_); hence *prob*(*I*_1_ + *d* ≥ *I*_2_) = 1. By the assumption, *min*(*I*_2_) ≥ *min*(*I*_1_). Hence, *min*(*I*_2_) + *d* ≥ *min*(*I*_1_) + *d* ≥ *max*(*I*_1_). Therefore, *prob*(*I*_2_ + *d* ≥ *I*_1_) = 1. Therefore, *prob*(|*I*_1_ – *I*_2_| ≤ *d*) = 1.

**Theorem 2** *Algorithm 6*, *the lazy timing correlation with a look-up table, is correct.*

Proof:

Let us first prove that the code block handling the left probe region is correct. The main idea of the look-up technique is that we can reuse the result of the timing condition (|@*e* – @*e_lookup_*| ≤ *d*, *ct*) in determining (|@*e* – @*e_new_*| ≤ *d*, *ct*). *e_new_* is the base tuple currently examined in the code and *e_lookup_* is the base tuple which was used for calculating *prob*(|@*e* – @*e_lookup_*| ≤ *d*) and stored in the look-up table where *e* is a tuple in the target stream buffer. The first line of the EfficientProbe function checks whether the tuple *e_new_* in the source stream buffer satisfies the condition *min*(@*e_new_*) ≤ *min*(@*e_lookup_*).

First, let us prove that it is always the case that *max*(@*e*) ≤ *max*(@*e_new_*) ≤ *max*(@*e_lookup_*). *L_L_*(*max*(@*e_new_*)) ≤ *max*(@*e_new_*) always holds. *e* is going to be used in processing *e_new_* only when @*e* belongs to [*L_H_*(@*e_new_*), *L_L_*(@*e_new_*)), i.e., *L_H_*(@*e_new_*) ≤ *max*(@*e*) < *L_L_*(@*e_new_*). Since the algorithm traverses the source stream buffer from the end of the unprocessed tuples, it should be always the case that *max*(@*e_new_*) ≤ *max*(@*e_lookup_*); hence *max*(@*e*) ≤ *max*(@*e_new_*) ≤ *max*(@*e_lookup_*). There can be two cases as shown in the following:

Case *min*(@*e*) ≤ *min*(@*e_new_*): In this case it is evident that *prob*(|@*e* – @*e_new_*| ≤ *d*) = *prob*(@*e* + *d* ≥ @*e_new_*) by Corollary 1. Similarly, *prob*(|@*e* – @*e_lookup_*| ≤ *d*) = *prob*(@*e* + *d* ≥ @*e_lookup_*). By Corollary 2 if (@*e* + *d* ≥ @*e_lookup_*, *ct*) is satisfied then so is (@*e* + *d* ≥ @*e_new_*, *ct*).

Case *min*(@*e*) > *min*(@*e_new_*): In this case @*e_new_* covers entire @*e*. Hence, *prob*(|@*e* – @*e_new_*| ≤ *d*) = 1 ≥ *prob*(|@*e* – @*e_lookup_*| ≤ *d*) by Corollary 3.

In both cases, it was shown that if (@*e* + *d* ≥ @*e_lookup,_* *ct*) is satisfied then so is (@*e* + *d* ≥ @*e_new,_* *ct*). The proof for the codes handling the right probe region is similar to this, hence is omitted.

## 5. Experiment and Analysis

In this section, we present experiment results showing various aspects of the proposed algorithms presented in the previous section. The data show that the lazy-family algorithms (lazy and lazy with look-up tables) give higher throughput than the eager algorithm; however they suffer from slower response time than the eager algorithm. We implemented a simple stream simulation system. Stream providers in the simulation system read the predefined event tuples and transmit them to the correlation algorithms. The implementation was done in Java. An Intel Xeon 1.8Mhz system with 1GB main memory on Windows XP professional was used for the experiment.

### 5.1. Experiment Results

**Figure 3.**
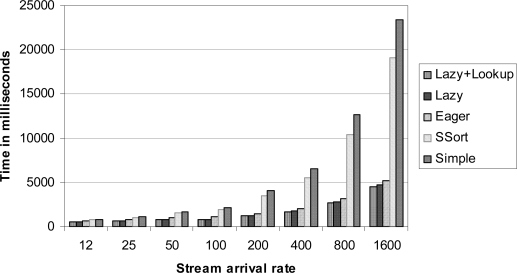
The execution times under various arrival rates.

**Figure 4.**
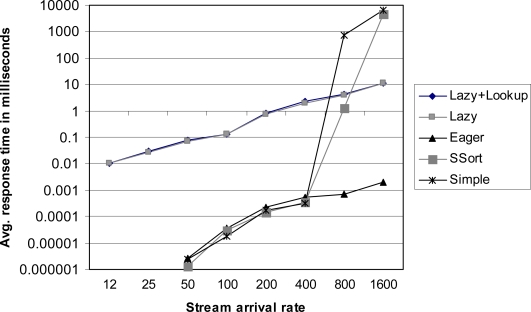
The average response times under various arrival rates.

We prepared the data files r12.dat, r24.dat, ..., and r1600.dat providing data streams of which arrival rates are from 12 tuples/second to 1,600 tuples/second respectively. We measured the execution times and the average response times of the correlation algorithms under these workloads. The execution time of an algorithm is measured by the total time spent by the algorithm. The response time is the summation of the response times for all tuples processed by the algorithm. The response time of a tuple (*e*_1_, *e*_2_) is computed by the correlation completion time minus MAX(max(@*e*_1_), max(@*e*_2_)). The results are shown in Figures 3 and 4. The growth of the execution times of the eager correlation and the lazy correlation family (lazy algorithm and lazy correlation with look-up tables) is much slower than those of the simple correlation family (simple correlation and simple sort correlation). The primary performance gain is achieved from the bounds analysis of satisfaction probability; the former algorithms save time by skipping the computation of the satisfaction probabilities for the tuples belonging to the satisfaction and violation regions. The simple sort correlation is faster than the simple correlation. This is mainly because the invalidation process is much effective in the simple sort correlation. By doing the correlation in bulk, the lazy correlation family shows better performance over the eager correlation.

It is also observed that the average response time of the eager correlation is better than that of the lazy correlation family. In addition, if the stream arrival rate is not so fast (until 400 tuples/second in this particular setting), the simple sort correlation and the simple correlation are better than the lazy correlation family as far as the average response time is concerned. The lazy correlation family intentionally delays the processing of incoming tuples; even in the case where the tuples can be processed right away; the tuples are waiting in the stream buffer until there are “enough” number of tuples. In contrast, the other algorithms process the incoming tuples as soon as they arrive. When the processing speed cannot catch up with the stream arrival speed, the response time begins to increase sharply.

The performance gain in the lazy correlation and the lazy correlation with a look-up table comes at the expense of larger memory usage and longer response time. Let us show the stream buffer usage of each correlation algorithm.

**Figure 5.**
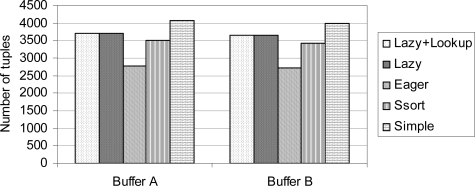
Average lengths of stream buffers.

Figure 5 shows the average lengths of stream buffers for the timing correlation algorithms. The length of the buffers for the eager correlation is the shortest. The buffer length for the simple sort correlation is shorter than that for the simple correlation. This is because the tuples are sorted in the simple sort correlation; hence, it is easier to find consecutive invalid tuples and remove them. In this experiment, we set the enough number of tuple of the lazy correlation to one thousand. Hence, roughly the length difference between the lazy correlation and the eager correlation is a thousand. Since the lazy correlation with a look-up table shares the same implementation of the lazy correlation except the probing part, the average buffer length is almost the same. Note that we also need to consider the size of the look-up table for analyzing the space requirement. The size of the look-up table can be as big as the stream buffers.

**Figure 6.**
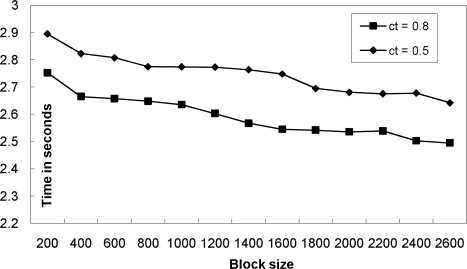
The execution times under different correlation block sizes.

Now let us show the effectiveness of increasing the block size determining the “enough” number of tuples to run the lazy correlation algorithms. Figure 6 represents the execution times under different correlation block sizes. In this experiment, we used the data file r500.dat (500 tuples/second) with the parameter *d* = 500. The experiments were done on two confidence threshold values 0.8 and 0.5. It can be observed that as we increase the correlation block size, the execution times tend to decrease, however, the differences are diminishing gradually.

**Figure 7.**
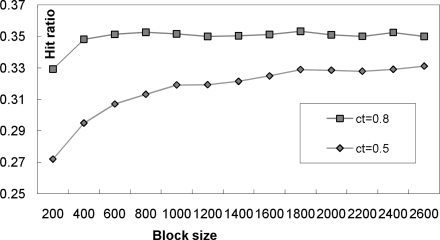
The hit ratio of the look-up table under different correlation block sizes.

Figure 7 represents the changes of the hit ratios of the look-up tables under different correlation block sizes. The hit ratio of the look-up table is computed by the number of tuples in the probe regions which did not need the probability computations divided by the total number of tuples in the probe regions. The look-up hit ratios were also gradually improved until they were stabilized at the correlation block size of 1,200 for this experiment. Note that the hit ratios largely depend on the property of the stream input, e.g., whether they are roughly sorted or not; thus, the hit ratios are not much changing in the figure. The maximum difference was observed at the transition from the correlation block size 200 to 400 on both cases.

**Figure 8.**
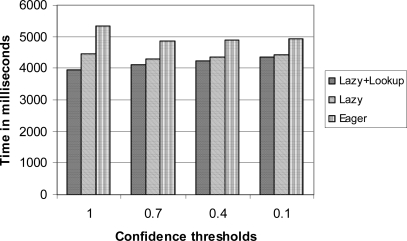
The execution times under various confidence thresholds.

Figure 8 shows the experiment results with the data file *seq.dat* where all tuples are ordered in their max timestamps. In this experiment, we set *d* = 1, 000 and *ct* = 1.0, 0.7, 0.4; and 0.1. It is shown that the lazy correlation with a look-up table performs the best in all cases. However the difference between the lazy correlation and the lazy correlation with a look-up table becomes smaller as we decrease the confidence threshold. This is primarily due to the small size of probe regions in low confidence threshold settings; thus the performance gain achieved by using the look-up table is not significant compared to the cases of the high confidence thresholds. We can explain this by the following analysis.

**Figure 9.**
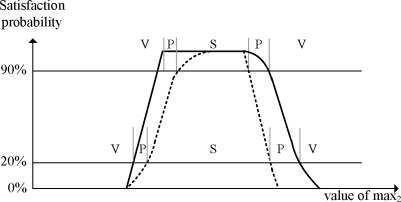
The interval timing correlation with high and low confidence thresholds.

It turns out the higher confidence threshold requirement, the larger left probe region as shown in Figure 9. In contrast, the lower confidence threshold requirement, the larger right probe region. An important observation is that the size of the left probe region is likely to be larger than that of the right one. The maximum size of the left probe region is *d* − *ρ*, which must be larger than that of the right region *π*. This is because *d* is much larger than *π* in typical cases. In the extreme case where *ct* = 100%, the right probe region does not exist while the size of the left probe region is maximized. In this specific experiment setting, the maximum size of the left probe region is *d* − *ρ* = 1, 000 − 20, and the maximum size of the right probe region is *π* − *ρ* = 200 − 20.

## 6. Summary

In this study, we proposed novel algorithms for the interval timing correlation. They can be used for extracting temporally related pairs of streaming sensor. In order to handle the uncertainty in timestamps, we adopted interval timestamps and included the confidence thresholds into timing conditions.

We extended a previously studied algorithm by adopting the approaches of the lazy evaluation and the result look-up. The lazy timing correlation also utilizes the upper-bounds and the lower-bounds. It postpones the evaluation until its re-evaluation condition is met and performs the correlation of the tuple blocks. In order to reduce the computation overhead of the satisfaction probability in probe regions, we added an idea of look-up technique. We measured the effectiveness of the proposed algorithms over the previous algorithms by comparing their performance under various workloads and presented the analysis. It turns out that the lazy family algorithms provide better performance with the sacrifice of extra memory for larger buffer and larger response time under slow streaming environment.

For the future work, the generalization of the proposed techniques for various probability distributions seems interesting. For the lazy approach, we need to derive upper-bounds and lower-bounds of the new probability distributions. The invalidation operations shown at the end of Algorithm 5 should be extended accordingly. For the look-up technique, we need an extension of the Corollary 2. Heterogeneous combinations of probability distributions may require non-trivial extensions. Similar changes are needed to the Algorithm 7.

It would be also interesting to apply the proposed techniques to the practical and real situations. As the sensor network become wide spread, we are looking forward to testing our algorithms in real life scenarios.
